# Development and testing of AAV-delivered single-chain variable fragments for the treatment of methamphetamine abuse

**DOI:** 10.1371/journal.pone.0200060

**Published:** 2018-06-29

**Authors:** Charles E. Hay, Guillermo A. Gonzalez, Laura E. Ewing, E. Elizabeth Reichard, Michael D. Hambuchen, Nisha Nanaware-Kharade, Sinthia Alam, Chris T. Bolden, S. Michael Owens, Paris Margaritis, Eric C. Peterson

**Affiliations:** 1 Department of Pharmacology and Toxicology, College of Medicine, University of Arkansas for Medical Sciences, Little Rock, Arkansas, United States of America; 2 Department of Pediatrics, The Children's Hospital of Philadelphia, Philadelphia, Pennsylvania, United States of America; 3 The Raymond G. Perelman Center for Cellular and Molecular Therapeutics, The Children’s Hospital of Philadelphia, Philadelphia, Pennsylvania, United States of America; 4 The University of Pennsylvania, Perelman School of Medicine, Philadelphia, Pennsylvania, United States of America; Scripps Research Institute, UNITED STATES

## Abstract

Methamphetamine (METH) substance abuse disorders have major impact on society, yet no medications have proven successful at preventing METH relapse or cravings. Anti-METH monoclonal antibodies can reduce METH brain concentrations; however, this therapy has limitations, including the need for repeated dosing throughout the course of addiction recovery. An adeno-associated viral (AAV)-delivered DNA sequence for a single-chain variable fragment could offer long-term, continuous expression of anti-METH antibody fragments. For these studies, we injected mice via tail vein with 1 x 10^12^ vector genomes of two AAV8 scFv constructs and measured long-term expression of the antibody fragments. Mice expressed each scFv for at least 212 days, achieving micromolar scFv concentrations in serum. In separate experiments 21 days and 50 days after injecting mice with AAV-scFvs mice were challenged with METH *in vivo*. The circulating scFvs were capable of decreasing brain METH concentrations by up to 60% and sequestering METH in serum for 2 to 3 hrs. These results suggest that AAV-delivered scFv could be a promising therapy to treat methamphetamine abuse.

## Introduction

Methamphetamine (METH) is a synthetic drug that causes periods of euphoria and stimulation, leading to very high abuse and a wide range of substance use disorders. These METH associated medical problems result in devastating socioeconomic consequences for individuals, families, and communities. METH acts at multiple sites in the brain, and research attempts to design small molecule receptor agonist or antagonists have not yet lead to any FDA-approved medications that can reduce relapse or craving.[1,2] Cognitive-behavioral therapy is a mainstay of METH addiction treatment, but is of limited help since most patients relapse within months after initial abstinence.[3,4] Because METH users are most vulnerable to recidivism during the first six to eight months of recovery, there is a critical need for pharmacological therapy to provide the user with support during this period.[1,5] A long-acting medication that could help protect against relapse, act synergistically with cognitive-behavioral therapy while not requiring frequent dosing could be ideal for improving medical outcomes.

Anti-METH anti-METH monoclonal antibodies (mAbs) act as pharmacokinetic antagonists to alter METH’s clearance, volume of distribution, elimination half-life (t_1/2_) and protein binding. In preclinical METH abuse models, mAbs can significantly reduce METH brain concentrations, slow the rate of METH entry into the brain tissue, shorten the duration of METH-induced locomotor activity.[6,7] These benefical effects could help to reduce or protect against the rewarding effects of METH use. Since our previous studies show that anti-METH mAbs are highly specific for METH-like drugs of abuse and do not appear to leave the blood stream when circulating through the brain, these mAbs are unlikely to bind to endogenous receptors.[8] These positive findings with anti-METH mAbs led to the development of a high affinity anti-METH chimeric mAb with a half life of 18 days in humans, which has completed a Phase 1a clinical trials.[9,10] These results further suggest the potential for using an immunotherapy to treat METH abuse.

Immunotherapies for METH can be classified into two broad categories: METH-conjugate vaccines and passive administration of anti-METH mAbs. For the vaccines, a METH-like hapten is linked to an immunogenic carrier for immunization of patients over a two to three month period.[2] Under ideal conditions this immunotherapeutic approach could offer sustained anti-METH antibody titers for 6 to 12 months, once immunological memory is achieved.[2] Potential disadvantages of this approach include the time required to achieve adequate antibody titers, highly variable patient immune responses, limited control of the affinity for METH, and poor immune responses in immune compromised patients (*e*.*g*., patients with HIV/AIDS).[2] In contrast, passive administration of preformed high affinity anti-METH mAbs offer immediate protection, do not have many of the limitations of active immunization, and should be relatively safe over a 6–18 month course of treatment.[11] The major disadvantage is the potential higher cost to patients, which could limit the availability to some patients. While the half-life of mAbs is relatively long, (~2 to 4 weeks in humans),[6] multiple doses would be required for a 8–12 month treatment period. Due to these limitations, even longer acting approaches that achieve sustained levels of anti-METH antibodies with less concerns about patient compliance could be advantageous to many addicted patients.

To increase efficacy and reduce the number of doses, we combined the advantages of gene therapy with mAb therapy. IgG proteins are the product of two genes, one heavy chain and one light chain and must undergo post-translational assembly, inter-chain disulfide bond formation, and glycosylation to function properly. The smaller IgG-derived single-chain variable fragment (scFv) protein consists only of the light and heavy chain variable regions of an IgG that are linked with a short protein sequence, and can be created by a single gene product that does not require complex posttranslational processing.[12] Because of the less complicated nature and smaller size, the scFv is more suitable for delivery by gene therapy. The scFv has multiple advantages in addition to being one gene product. These include a lower production cost and requiring less protein than full IgGs to neutralize METH. In rats, anti-METH scFv6H4 (K_D_ = 4 nM for METH) quickly increases METH serum concentrations, and can form multivalent forms (dimers, trimers, etc.) independent of METH binding that have a t_1/2_ of about 4 hours.[12] While this rapid clearance has clinical potential as a treatment for METH overdose, increasing the serum t_1/2_ of the scFv would result in a beneficial, longer-lasting efficacy for chronic addiction therapy.

We hypothesize that a viable method to overcome the relatively short half-life of scFvs and full IgG mAbs is to use a gene therapy approach to induce a constant infusion of the scFvs into the host’s circulation by packaging plasmids into a non-pathogenic carrier, such as adeno-associated virus (AAV). After the gene of interest has been packaged into the virus capsids, the viral coat is able to insert the gene sequence into the nuclei of host cells. The host cells are then able to express the gene of interest. There have been many studies that have utilized AAV8 such as clinical trial data for both hemophilia[13,14] Recently, the FDA has approved for clinical use the first gene therapy to treat biallelic RPE65-mutationassociated retinal dystrophy.[15] This suggests that a gene therapy using an AAV8 delivery system could confer similar long-term expression of therapeutic antibodies. AAV8 has been shown to have tissue tropisms for mouse liver, smooth muscle, heart, kidney, brain and pancreas. [16–18] AAV8 has been shown to result in relatively high protein expression in the liver, which is well known to secrete numerous proteins into the circulation. [16,18,19] Further, AAV serotype 8 was selected since it has been shown that, compared to other AAV serotypes, there is a lower prevelance of preexisting anti-AAV neutralizing antibodies in humans.[20] Recent reports also show that AAV therapy is a potentially viable approach for delivery of anti-drug IgGs for nicotine, cocaine, and METH abuse. [21–24]

In this report, we present the design of two novel anti-METH scFv plasmids and subsequent packaging into AAV8 vectors. We compared the in vivo scFv production and immunochemical characteristics of scFv in mice with scFv protein standards, measured long-term expression of AAV-scFvs, and characterized the disposition of METH in mice treated with AAV-scFvs.

## Results

### Design and generation of scFv plasmids and AAV8 capsids

**Fig 1:**
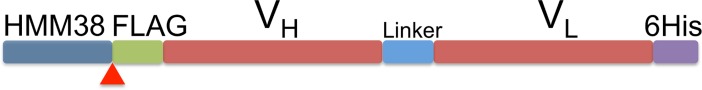
Schematic of the prototype scFv design. V_H_, variable heavy region; V_L_, variable light region; Linker, 15 amino acid linker; His6, 6-histidine tag for purification and identification; FLAG, FLAG tag for identification; HMM38, a secretory signal sequence. The HMM38 at the 5’ end of the sequences is cleaved during secretion at the site indicated (triangle).

The scFv cDNA constructs (Fig 1) were cloned into pscAAV-GFP, a self-complementary vector. Self-complementary vectors have the advantage of higher efficiency since they do not need to be converted to double stranded DNA prior to transcription. AAV8 capsids can contain up to 4.7 kB of DNA of a single stranded vector or up to 2.2 kB in a circular plasmid.[25] Since our scFv sequences are less than 1000 base pairs, it met the <2.2 kb criteria and thus allowed us the opportunity for possibly much higher expression than a single stranded vector.

**Fig 2:**
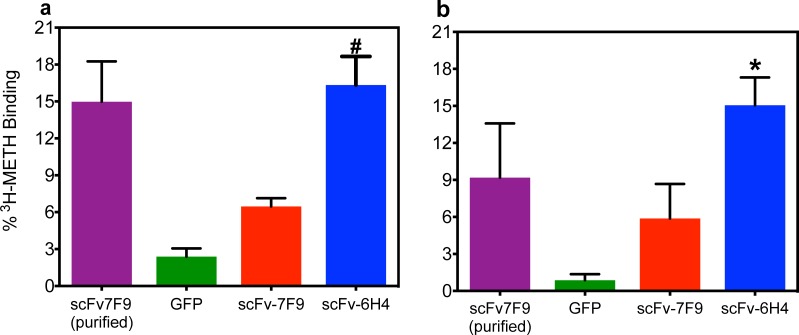
Hydrodynamic AAV-scFv plasmid transfected mice expressed functional anti-METH scFv-6H4 and scFv-7F9. ^3^H-METH binding was determined by equilibrium dialysis. Purified scFv7F9 (4 μg/ml) was used as a positive control. ScFv6H4 transfected serum samples bound ^3^H-METH significantly higher than GFP (negative control, nonspecific serum binding) [#, p < 0.001; *, p < 0.01] at 24 and 48 hrs post-AAV8 treatment. ScFv7F9 also bound more, but was not statistically significant. (b). Points are shown as mean ± SEM (n = 3 per group).

A hydrodynamic delivery study (rapid i.v. delivery of DNA) with the scFv plasmids was performed to ensure scFv expression, secretion, and function in serum. Mice were administered plasmids of scFv7F9, scFv6H4, or GFP via tail vein and then sacrificed at either 24 or 48 hrs post-injection. Serum samples were analyzed via rapid equilibrium dialysis (RED) to determine METH binding capability. Fig 2A shows the scFv6H4 group, but not the scFv7F9 group, exhibited significantly greater levels of binding to ^3^H-METH over GFP (p < 0.01) at 24 hrs post-injection. At 48 hrs post-injection, there was a significant difference between GFP and scFv6H4 groups but not between GFP and scFv7F9 groups (Fig 2B).

### Long-term expression of AAV-scFvs

**Fig 3:**
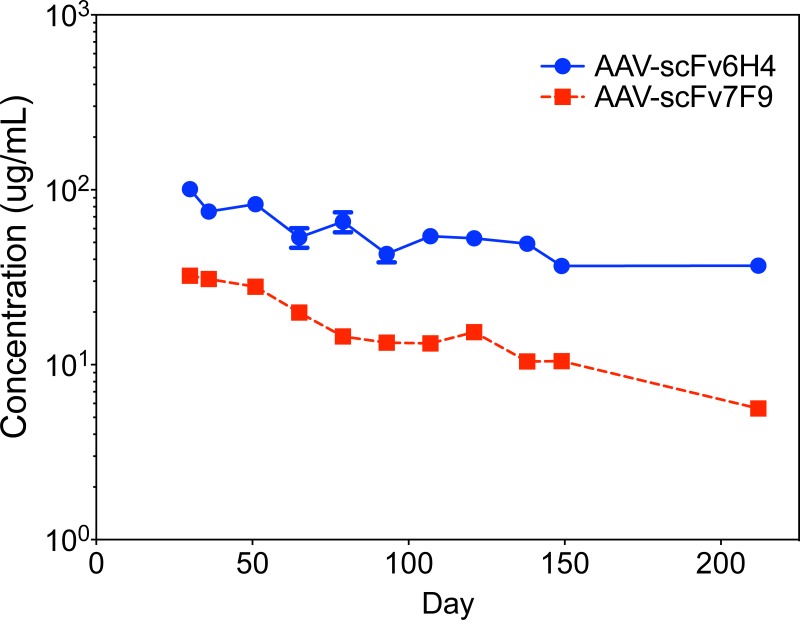
Expression levels of AAV-scFv constructs over time. AAVscFv-6H4 and AAV-scFv7F9 serum relative expression-time profile in AAV-scFv injected mice as measured by functional ELISA. Serum concentrations showed expression of scFv through day 212. Points are shown as mean ± SEM (n = 10 per group).

Since the administration of plasmid DNA demonstrated *in vivo* an expression of each scFv, we generated AAV serotype 8 for each of scFv constructs. AAV-scFv serum titers and duration of scFv expression were then measured in mice. Fifteen days following intravenous injection of 1 x 10^12^ vector genomes (vg)/mouse dose of AAV-scFv (day 0), scFv6H4 or scFv7F9 was detected in serum, with measurable scFv titers continuing through the final time point, day 212 (Fig 3). Peak expression for scFv6H4 occurred on day 30 at 101 μg/ml. In contrast, scFv7F9 reached a maximum concentration in serum of 89 μg/ml on day 15.

### Comparing scFv protein affinities to AAV-expressed scFv affinities

**Fig 4:**
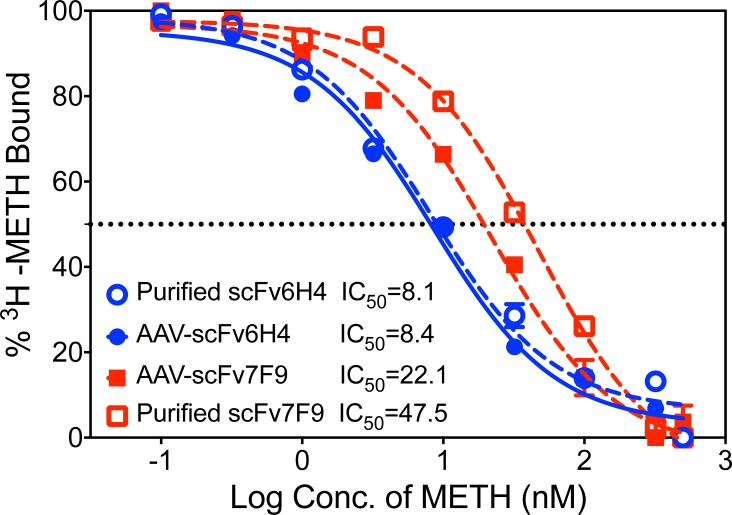
Comparison of IC_50_ values for METH between culture-produced scFv and expressed AAV-scFv. A competitive binding assay was performed with each of the variants. The IC_50_ (in nM) for both scFv and AAV-scFv variants was estimated at 50% ^3^H-METH bound (dotted line). Individual data points are shown as the mean ± SEM (n = 8 per group).

The IC_50_ of AAV-expressed scFv6H4 and scFv7F9 to METH was compared to that of each scFv produced *in vitro*.[12] Titration binding assays were first conducted to normalize each protein sample to a concentration that binds 20% of 50,000 dpm of ^3^H-METH to reduce ligand depletion effects. These normalized scFv concentrations were used in competition-binding assays to determine the IC_50_ of both AAV-expressed and *in vitro* produced scFv6H4 and scFv7F9. AAV-scFv6H4 and *in vitro* scFv6H4 had nearly identical IC_50_ values at 8.4 nM and 8.1 nM, respectively (Fig 4). In contrast, the expressed AAV-scFv7F9 form (IC_50_ = 22.1 nM) showed over two-fold greater affinity for METH than scFv7F9 expressed and purified from Chinese Hamster Ovary (CHO) cells (IC_50_ = 47.5 nM). While the AAV-scFv7F9 expressed antibodies had over twice the affinity to METH as the *in vitro* expressed scFv7F9, the two affinities were not significantly different from each other.

### Disposition of METH and AMP after AAV-scFv treatment

**Fig 5:**
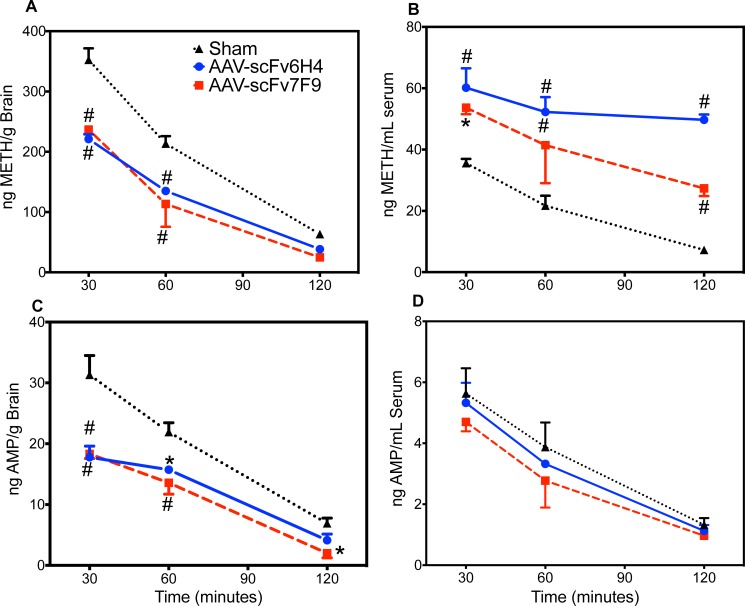
A comparison of METH or AMP brain and serum concentrations over time, after a 0.56 mg/kg *ip* injection of METH, between AAV-scFv6H4, AAV-scFv7F9, and a saline control at day 21 post AAV8 administration. Mice treated with either AAV-scFv6H4 or AAV-scFv7F9 showed significantly lower brain METH concentrations (a) and significantly higher serum concentrations of METH (b) than the saline-treated mice (*, p < 0.05; #, p < 0.001). There was also a significant decrease in AMP brain concentrations (c) in the AAV-scFv treated groups compared to control mice but no difference in serum AMP concentrations (d). Points are shown as mean ± SEM (n = 3–4 per group).

The activities of both scFvs were determined by measuring changes in both serum and brain concentrations of METH over a 120-min time-period after a single intraperitoneal (*ip)* injection of METH. On day 21 post-administration after receiving a 0.56 mg/kg METH *ip* injection, mice expressing either AAV-scFv6H4 or AAV-scFv7F9 maintained significantly higher serum METH concentrations compared to sham mice (p<0.001) and significantly lower concentrations of METH in the brain (p<0.05) at each time point throughout the 2 hr experiment (Fig 5). This suggested significantly less METH diffused across the blood brain barrier from the vasculature to the brain when compared to sham mice. Our AAV-scFvs showed very low affinity for amphetamine, an active metabolite of METH ((AMP), K_D_ = 2.1 μM for scFv7F9 and K_D_ = 48.8 μM for scFv6H4). We found that the concentration of AMP in the brain was significantly lower in the AAV-scFv6H4 and AAV-7F9 treated mice compared to control mice at 30 and 60 min and significantly lower in AAV-scFv7F9 mice compared to control at 30, and 60 min. However, there were no significant differences between control and AAV-scFv treated mice for serum AMP concentrations.

**Fig 6:**
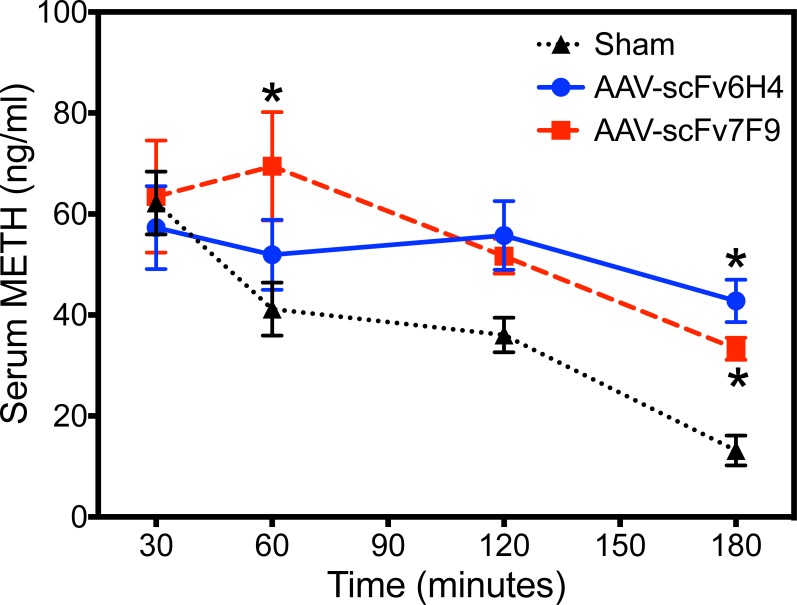
Pharmacokinetic study. Fifty days after AAV-scFv treatment, mice were injected *sc* with 1 mg/kg METH. Blood samples were collected at 30 and 120 or 60 and 180 min after METH injection. LC-MS/MS was used to determine serum METH concentrations. Because samples were not collected from every mouse at every time point, a non-repeated measures two-way ANOVA was used to analyze the METH concentration-time data. There were significant differences between control and AAV-scFv7F9 treated mice at 60 min post injection and between sham and both AAV-scFv groups at 180 min (* p < 0.05). Data points are shown as mean ± SEM (n = 5–6 per group).

**Fig 7:**
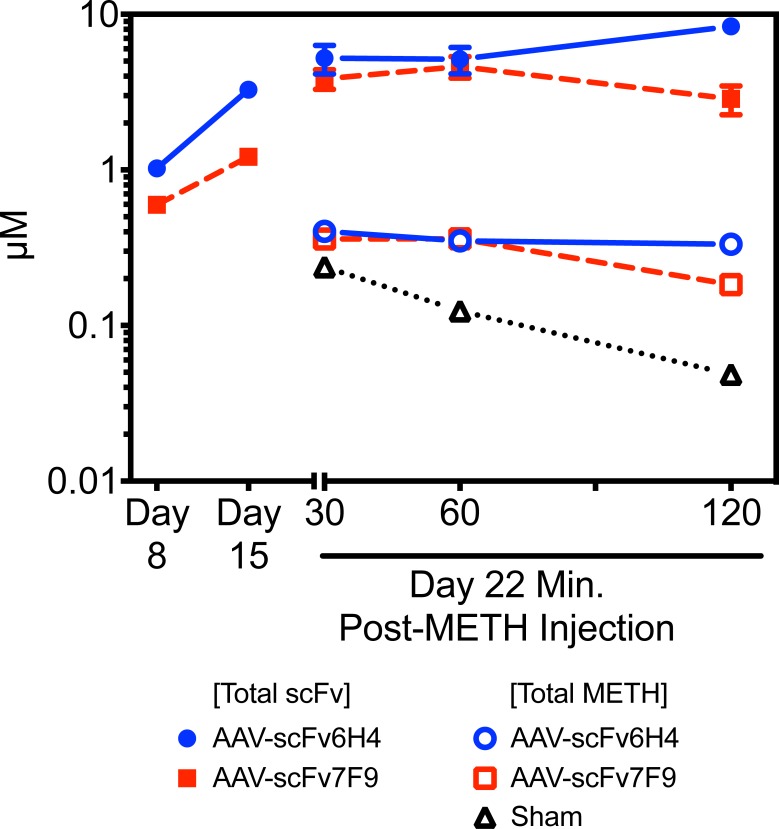
Comparison of METH and scFv molar concentrations over time. On day 0, mice were injected with PBS (control treatment), AAV-scFv6H4, or AAV-scFv7F9. Serum scFv6H4 and 7F9 concentrations were measured via ELISA on days 8 and 15. On day 22, mice were treated with METH as described in Fig 6. Both serum scFv and METH concentrations were measured. Mice treated with either AAV-scFv6H4 or AAV-scFv7F9 showed significantly higher serum concentrations of METH than the control mice (*, p < 0.05; #, p < 0.001). Data points are mean ± SEM (n = 3–4 per group).

In a separate experiment to test the activity of the AAV-scFvs longer-term effects, mice expressing either AAV-scFv6H4 or AAV-scFv7F9 on day 50 post-AAV8 treatment showed significantly higher levels (p<0.001 and p<0.05 respectively) of METH being sequestered in the serum at both 60 and 180 min for scFv7F9 and 180 min for scFv6H4 after injection of 1 mg/kg METH subcutaneous (*sc)* when compared to sham mice (Fig 6). After converting the scFv and METH concentrations, in the serum, from the disposition studies to their molar concentrations (Fig 7), the molar concentrations of the scFv appeared nearly 10 times greater than that of METH at all three time points. Both the scFv6H4 and the corresponding METH concentrations remained relatively constant over the two hrs suggesting these scFvs were sequestering that METH in the blood stream.

### Animal health

During all studies, mice were weighed every other week. Mice in all groups exhibited similar rates of weight gain through the duration of the studies, with no significant differences between control and AAV-scFv treated groups (S1 Fig). During the long-term expression experiment, we measured three biochemical markers of liver and kidney health status, alanine aminotransferase (ALT), aspartate aminotransferase (AST), and creatinine (S2 Fig). There were significant differences in ALT levels between sham and AAV-scFv7F9 at day 138, in AST between sham and AAV-scFv6H4 at day 149, and in creatinine between sham and AAV-scFv6H4 at day 121. However, biochemical markers remained within the normal range. We also measured internal organ weight of the AAV8 mouse cohorts at the end of the long-term expression study, and compared AAV-scFv treatment groups to the sham cohort (S3 Fig). The livers of the AAV-scFv6H4 group showed a small but significant mass increase at the end of the 212 day study (p < 0.01).

## Discussion

In this article, we present the design and *in vivo* characterization of two potentially therapeutic anti-METH mAb-based gene therapies. The results of these experiments support our hypothesis that using an AAV8 delivery system to induce *in vivo* expression of anti-METH scFvs can dramatically extend the duration of protection compared to *iv* delivery of single bolus doses of anti-METH scFv or IgG. A single injection resulted in months of sustained serum antibody concentrations compared to days (in mice) to weeks (in humans) of an injection of IgG. Serum concentration and duration of action studies showed that both AAV-scFvs were continuously expressed until the study was ended at 212 days. Compared to previous studies showing the METH protection duration of a single bolus dose of scFv (30 min) or of IgG (6–8 days) in mice,[12,26,27] AAV-scFv delivery dramatically increases the duration of protection by almost 28-fold. In terms of treatment potential, a single dose of AAV-scFv could potentially negate the need for repeated doses of scFv or IgG protein, thus signicantly reducing the need for patient compliance with repeated medication dosing.

This long-action of AAV-induced expression can be compared to a long-term continuous infusion, thus standard pharmacokinetic (PCKN) principles can be used to estimate the rate of infusion/expression of scFv. Because the scFv protein sequences were not changed from those in earlier scFv PCKN studies from our laboratory, the clearance rate (Cls) should remain the same at 2.0 ± 0.04 ml/min/kg.[12,26] From this known Cls parameter, and utilizing the pharmacokinetic equation for the steady state concentration (C_SS_) of a drug ($C_{SS}=\frac{inf}{Cls}$), the infusion rate (inf) can be determined for each scFv. We used the average circulating concentrations after reaching a near-steady state of AAV8-scFv6H4 (68.0 ug/ml) and AAV8-scFv7F9 (28.4 ug/ml) for our C_SS_ values. Considering the average weight of the mice in the studies (25 g), we were able to calculate the mean infusion rate of our therapy to be apporixmately 2.5 μg/min for AAV-scFv6H4 and 0.6 μg/min for AAV-scFv7F9.

It is important to note, that while there appears to be a continuous infusion, the rate of expression of these anti-METH scFvs was not uniform. During these studies we learned that the expression of the scFv is not constant throughout the study. Indeed, the expression of the scFv requires about three weeks to reach peak expression levels. During this time, we expect that protection against METH would be proportional to expression levels. Our scFv expression plot (Fig 3) shows expression levels peak around 21 days post-AAV administration. Thus constant expression does not imply a uniform expression rate, as it can be observed that the expression of the scFv slightly oscillates and slowly decreases over time. The gradual decrease in expression over time is expected, as AAV does not replicate, and expression ends when the host cell dies. The oscillation we observed could be the result of experimental variation, but we do not think this is the case as we have observed it in every group of mice treated with these AAV-scFv therapies over the past 4 years (4 groups of 8–10 mice for each scFv). It is much more pronounced for the higher-expressing scFv6H4. To the best of our knowledge, there have been no other studies that have noticed this oscillation trend in expression levels.

In terms of protection against METH effects, significantly greater concentrations of METH were sequestered in the serum while less METH entered the brain over time (Figs 5 and 6). This suggests that the AAV-delivered scFv were effective at maintaining METH brain concentrations about 27–60% lower in the AAV-scFv treated mice than in the controls. Due to the study design and sample limitations, we were not able to correct for the differences in METH concentration in the brain vasculature system with and without antibody binding, thus these values likely underestimated the level of METH protection in the presence of scFv. Mice treated with the AAV-scFv treatment showed 1.5 to 6.8 fold higher METH concentrations in the serum than the sham mice, providing evidence that the METH is being sequestered in the serum and reducing further METH entry into the brain. The AAV-scFv therapy showed similar decreased amounts of amphetamine, the major active metabolite of METH, into the brain but not in the serum (Fig 5C and 5D). Interestingly, the relative patterns of declining serum METH concentrations follow similar patterns for both AAV-scFvs as well as the controls. The relative differences in the METH serum concentration, but not brain concentration, over this time period might be due to the individual pharmacokinetic properties of the scFvs. In addition to the monomer formation, scFv6H4 forms spontaneous dimer and trimer configurations,[12] which increase its size and increases its half-life in the serum. In contrast, ScFv7F9 does not appear to form these higher order structures and thus has a much shorter half-life. [26,28] The relative reduction of METH concentration at the 120 min time point could reflect faster METH clearance associated with scFv7F9. The PK difference in these two scFvs was one of the reasons we chose to test both constructs.

Interestingly a similar study with an AAV delivered anti-METH IgG using the same variable sequence as scFv6H4 showed conflicting serum results to those we observed.[23] In this study, they reported a 23% decrease in METH in the serum of their treated mice compared to mice administered PBS 10 minutes after a 1 mg/kg METH ip dose. This is an unusual observation, as antibodies against drugs of abuse have been shown to have small volumes of distribution and partition their targets in the serum.[21,22,29–37]

For our studies, we chose two routes of METH administration to determine if the AAV8-scFvs could affect METH distribution via 2 routes of administration. The first experiment, conducted on day 21 of expression was a lower 0.56 mg/kg *ip* dose, meant to model an oral dose which is subjected to liver first pass effect and the metabolizing P450 enzymes in the liver (primarily CYP2D6 for METH)[38]. We then chose a higher dose of METH (1 mg/kg) later in the expression period (50 days) to present a greater drug challenge to the scFv therapies after an extended time period. We chose to deliver METH via an *sc* route, which results in shorter serum T_max_ and higher C_max_ of METH, thus a greater challenge to the potential therapy. Thus, the important comparisons are between the AAV-scFv treated groups and the sham (no AAV-scFv) groups *within* each experiment.

An earlier study from our lab group using *iv* bolus doses of IgG anti-METH mAbs showed greater differences in the disposition of METH in the brains and serum of rats when compared to controls than we observed in these studies.[32] These studies showed that after a 503 mg/kg mAb dose *iv*, there was a 30-fold increase in METH concentrations in the serum of rats, starting at 30 min and increasing over time. Starting at 30 min post METH administration, there was a 60% decrease in METH concentration in rat brains over controls. The dose used for the previous study was calculated to be equimolar mAb METH binding sites to a 1 mg/kg dose of METH. In comparison, in a 75 kg human, these results would require a 37.7 g dose of mAbs. AAV-delivered gene therapy has the potential to overcome these limitations Although our study used a high AAV dose of 1 x 10^12^ vg/mouse (equivalent to 4 x 10^13^ vg/kg), it is within the range used in current human clinical trials. For instance, an ongoing Phase 1/2 clinical trial for adults with moderate to severe hemophilia B (NCT02618915) proposes 1 x 10^13^ genome copies/kg of AAV rh-10/FIX as a highest possible dose. Importantly, sustained coagulation Factor VIII expression in hemophilia A patients has been achieved with doses up to 6 x 10^13^ vg/kg, when combined with glucocorticoids.[39] Clearly, our goal is to produce a therapy with a significantly lower efficacious dose range in future iterations of this therapy, potentially feasible by modifications in the AAV vector and/or the transgene cassette.

The AAV-scFv dose delivered (1 x10^12^ vg/mouse) resulted in lower levels of METH entry into the brain, but even at the tested 0.56 mg/kg METH dose, there was only a reduction in METH brain concentration of 27–60%, when compared to control mice. However, we think that the 40–60% reduction in brain METH concentration could result in reduced METH-induced behavioral effects. For example, previous studies from our group[40] have shown that methamphetamine-induced locomotor effects in rats after administration of a high affinity anti-methamphetamine monoclonal antibody, that a 37% drop in brain METH correlated with a 31% decrease in METH-induced locomotor duration after a 1.68 mg/kg METH, 28 days after mAb7F9 administration. Although this remains to be determined with our AAV treatments, we think that we will see similar locomotor effects in mice.

Other studies attempted to treat other drugs of abuse with gene therapies using full IgG antibodies rather than scFvs, and observed circulating antibody concentrations as high as 1 mg/ml after a 1x10^11^ vg/mouse dose.[9,21,35] Taking into account the differences in size and the number of antigen binding sites, their observed peak circulating concentrations are about ~3.5x higher than the AAV-scFv6H4 expressing mice at their peak. This increase in circulating concentrations are likely due to the significant differences in the clearance rates of the scFvs and IgG antibodies. Many studies report that full length IgG antibodies can have a half-life of up to a week in rodents[33,41,42] whereas, as stated above, scFv7F9 has a half-life of up to 1.3 hrs in rats. We believe that this large decrease in half-life of the scFvs is a likely culprit for the observed lower circulating concentrations.

For this study, two different scFv constructs were used scFv6H4 and scFv7F9, with the primary differences being in their sequences of the three complimentarity-determining regions (CDRs).[43] As can be seen in Fig 3, scFv6H4 was consistently detected at roughly 2.5-3x higher concentrations than scFv7F9, yet in Fig 5A and 5B the disposition of METH in the brains and serum samples of the mice are similar for the two scFvs. Considering this, AAV-scFv6H4 was likely in higher concentrations due to its ability to form multimers with itself and thus have a longer half-life. This polymerization was able to increase scFv6H4’s half-life from 6 min as a monomer to 228 min as a multimer.[12] While the half-life of the monomer version of scFv6H4 is drastically lower than the 78 min half-life of scFv7F9, the multimer version of scFv6H4 has a half-life that is nearly three times that of scFv7F9. This is about the average difference in circulating concentrations between scFv6H4 and scFv7F9. The similarities in *in vivo* functional effects (Figs 5 and 6) were possibly due to *in vivo* inactivation of the scFv6H4 binding site.[42]

As can be observed in Fig 7, there is a substantially larger total molar concentration of the scFv protein compared to the total molar concentration of METH. When considered with the data presented in Figs 5 and 6, this suggests that while there may indeed be greater molar equivalents of the scFv, not all the scFv proteins are likely occupied with METH. This further suggests that the 6-min half-life of scFv6H4 may be detrimentally impacting the neutralizing ability of the AAV-scFv6H4 construct. Alternatively or in conjunction, the scFv rate of synthesis and infusion in the circulation may be a limiting factor. This explanation could also potentially explain the lower than expected neutralizing ability of AAV-scFv7F9. Therefore, this therapy, in its current form, may not be able to provide clinically relevant protection against METH dosing. We have hypothesized that there are two options to increase the level of possible protection: increase the viral copy number injected or decrease the clearance rate of the antibody fragments once expressed. Increasing the injected viral copy number may elicit an immune response against the viruses as seen in hemophilia patients. [14,39] Although this may be managed by glucocorticoids, this risk makes it the least favorable option. Moreover, it can drastically increase production costs per injection. Alternatively, novel ways to decrease the clearance rate of the expressed antibody fragments can be implemented through either conjugating a sequence to the scFv or to use different antibody variants, which possess more favorable pharmacokinetic properties. [44,45]

A concern of any new therapy is the potential for adverse side effects. Since the AAV-delvered genes were designed to be produced in the liver, we assessed biomarkers of overall health of the mice (S1–S3 Figs). The serum chemistry biomarkers ALT, AST, and creatinine, as well as mouse body weights and organ weights, were measured throughout the second half of the duration of expression study for the AAV8 and sham animal cohorts. We observed transient elevations of liver enzymes (S2 Fig) but remained within the normal ranges of enzymes that we could find in the literature. Our preliminary safety results suggest that the AAV-scFv treatments do not negatively affect mouse health. Although our sham group did not receive a control vector, we do not think it is likely that the AAV administration at the doses used here could negatively impact animal health. It is worth noting that even larger doses of AAV8 (1.25 x10^14^ vg/kg) have been administered in animals, with no effects on their health.[46] Of course, future in-depth toxicity studies will be required to take a more comprehensive picture of the effects of anti-METH AAV treatments both short- and long-term, as well as the ability of these treatments to prevent METH-induced toxicity, such as neuroinflammation and effects on the Blood-Brain Barrier.

In conclusion, these studies show the design, production, and preclinical characterization of high-affinity therapeutic AAV-scFv6H4 and AAV-scFv7F9 against METH. Both AAV-scFvs were expressed for at least seven months in mice, retained high affinity for METH, and were able to significantly redistribute METH and the active metabolite, amphetamine from the brain into serum. These first, proof-of-concept, preclinical studies suggest that AAV-mediated gene transfer of anti-METH scFvs could potentially be a viable approach to significantly impact the treatment of METH abuse.

## Methods

### Animal usage

All protocols and experiments used were performed in accordance with and approved by the University of Arkansas for Medical Sciences Institutional Animal Care and Use Committee. Adult, male BALB/c mice were purchased from Charles River Laboratories (Raleigh, NC) and housed in groups of 4–5 in a light-controlled environment (12 hr light/dark cycle). They received water and food pellets *ad libitum*, maintaining body weights between 20–33 g. Mice were randomly selected for all experimental groups.

Animal health was monitored by recording weights weekly and by measuring liver- and kidney-specific serum chemistry markers. We measured alanine transaminase (ALT), aspartate transaminase (AST), and creatinine via enzymatic microplate assays (Enzychrom Aspartate Transaminase Assay Kit and Enzychrom Alanine Transaminase Assay Kit, BioAssay Systems, Hayward CA; Mouse Creatinine Assay Kit (Enzymatic), Crystal Chem, Inc., Downers Grove IL). At the end of each study, brain, liver, kidneys, heart, and spleen were harvested from each animal and weighed and stored at -80°C for further analysis.

### Drugs and reagents

All METH used, both unlabeled and tritium-labeled, was obtained from the National Institute of Drug Abuse (Bethesda, MD). Chemicals and other reagents were purchased from Fisher Scientific (Pittsburgh, PA) or ThermoFisher Scientific (Waltham, MA) unless otherwise stated. Plasmid and protein production were performed using Qiagen kits (Valencia, CA). All chemicals used in the LC/MS-MS analysis were LC/MS grade materials purchased from Fisher Scientific. Methamphetamine and amphetamine standards were purchased from Cerilliant (Round Rock, TX). Different lots of METH and AMP were used for standard curve samples and quality control samples. Manifold, SPE columns, and injection vials for LC/MS-MS analysis were purchased from Phenomenex (Torrance, CA).

### Design and generation of scFv plasmids and AAV8 capsids

Both scFvs (scFv6H4 and scFv7F9) were derived from anti-METH mAbs generated previously.[6,12] The scFv6H4 sequence is available in Genbank (accession # ACO48884) and the sequence of mAb7F9 is reported in US Patent 9023353. [12,47] The expressed fragment of the antibodies were comprised of the variable heavy chains (V_H_) and variable light chains (V_L_) of the respective scFvs connected by a 15 amino acid linker. These sequences were flanked by a flag (FLAG) sequence at the 5’ end and a 6-histidine tail (6HIS) at the 3’ end. Also expressed was a cleavable signal secretion sequence (HMM38), to ensure correct folding and routing through the cellular secretory pathway.[48] As can be seen in Fig 1, the order of the elements in each scFv is as follows: 5’–HMM38 –FLAG–V_H_−Linker–V_L_− 6HIS– 3’. Plasmids were cloned into cassettes with a hepatic control region (HCR), a human α-1 anti-trypsin promoter (HAAT), and an SV40 intron prior to the HMM38 sequence. The scFv cDNA constructs were cloned into pscAAV-GFP (# 32396, Addgene, Cambridge, MA). Standard DNA cloning techniques were used and all plasmids were maintained in *E*. *coli* strain DH5α. Sequences of all plasmids were confirmed by sequencing at the University of Arkansas for Medical Sciences DNA Sequencing Core Facility. To prepare plasmids for *in vivo* testing and AAV8 production, plasmids were replicated in *E*. *Coli* and purified by Qiagen endotoxin-free plasmid purification kits. DNA purity and concentrations were determined by UV_260/280_ spectrophotometry and gel electrophoresis.

To ensure the DNA plasmids elicited functional and properly folded proteins, a hydrodynamic infusion was performed. Plasmids were suspended in phosphate-buffered saline (PBS), and 5 μg of plasmid DNA (10% w/v of total mouse weight) containing functional green fluorescent protein (GFP), scFv6H4 or scFv7F9 were administered to male BALB/c mice via tail vein over 10–15 sec. Afterwards, mice were returned to their cages. Blood samples were collected at 24 and 48 hrs post-injection and analyzed via rapid equilibrium dialysis (RED) devices as described in Thakkar et al.[49] Briefly, RED devices were loaded into a base plate, in duplicate, and each red side of the device was loaded with 90 μL containing 50,000 DPM ^3^H-labeled methamphetamine and 10 μL of sample. The white side of the device was loaded with 300 μL of PBS. The RED devices were covered and allowed to equilibrate overnight on an orbital shaker. After equilibration, 50 μL were removed from the red and white sides and added to their respective scintillation vials containing 4 mL of ScintiVerse BD cocktail (#SX18-4, Fisher Scientific). Radioactive content was measured with a Tri-Carb 2910TR Liquid Scintillation Counter. Percent bound was calculated as (DPM red side–DPM white side)/(DPM red side) x 100%.

After confirmation of proper expression from the hydrodynamic expression study, DNA sequences were packed into AAV8 capsids by SAB Tech (Philidelphia, Pennsylvania), whom employs a helper virus-free, triple plasmid transfection method for AAV8 vector production. The plasmids coding for the scFv’s, mini-adenovirus plasmid, and a plasmid coding for an AAV8 helper plasmid were transfected into HEK 293 cells at equal ratios to each other.[50] The RVC then used roller bottle-based tissue culture and transfection to grow the AAV-scFv vectors on a large-scale.[51]

### Long-term expression of AAV-scFvs

Thirty male, BALB/c mice were randomly assigned to one of three groups. Experimental groups received 1 x 10^12^ vector genomes (vg) per mouse of either AAV-scFv6H4 or AAV-scFv7F9 in 5% sorbitol/1x PBS for a total volume of 100 μL. The control group mice were given an equal volume of PBS/5% sorbitol. The treatments for all three groups were administered via tail vein injections on week 0. Blood sampling was performed every other week beginning at week two. Blood samples were incubated for 2 hrs at 21^o^ C, and then centrifuged at 12,000 x g for 10 min. Serum was collected from the resultant supernatant and stored at -80^o^ C for analysis.

Serum samples were thawed on ice and used for functional ELISAs. A 96 well Immulon 4HBX plate (#14-245-193B, Thermo Scientific) was coated with 50 μl of 2.5 μg/ml of a BSA-METH hapten in a 0.2 M sodium bicarbonate buffer. The plate was incubated at room temperature (~21^o^ C) for one hour. The wells were washed three times with 100 μl of 1x PBS + 1% Tween 20 and placed on an orbital shaker for 3 min and aspirated. One hundred eighty μL of Superblock (Thermo Scientific, Waltham, MA) were added to each well and placed on an orbital shaker to incubate for 1 hr. After removing the Superblock solution from each well, the plate was washed three times as before, and then 50 μl of standards and serum samples were added to their respective wells. The plate was incubated on an orbital shaker for one hr at room temperature. Standards were made from primary stocks of each purified scFv protein and then serially diluted at a 1:2 ratio to produce a standard curve with concentrations from 3.125 to 400 ng/mL. The standard curve of purified scFv protein was included on each plate for accurate serum scFv concentration determination. Serum samples were diluted 1:250 and 1:500 for AAV-scFv6H4 samples and 1:100 and 1:200 for AAV-scFv7F9 samples. After incubation, the plate was washed three times, and 50 μl of 0.25 μg/ml 6X-His Epitope tag HRP-conjugated antibody (#652504, Biolegend, San Diego, CA) in Superblock were added to each well and incubated at room temperature on an orbital shaker for 1 hr. The plate was then washed three times. To start the colorimetric and enzymatic reaction, 50 μl of 1 Step Ultra TMB (Thermo Scientific) were added to each well and incubated for 2 to 3 min. At the end of the incubation, 50 μl of 2 M H_2_SO_4_ were added to each well to stop the enzymatic reaction. Absorbance at 450 nm was measured on a Biotek HT Synergy plate reader (Biotek, Winooski, VT).

### Comparing scFv protein affinities to AAV-expressed scFv affinities

A competition-binding assay was also performed as previously described.[49] Briefly, this assay used RED devices in duplicate, and labeled and unlabeled METH to determine the IC_50_ of the scFvs to METH. Based on the results of a titration-binding assay, scFv antibodies were diluted to concentrations that bind 20% of 50,000 DPM ^3^H-METH. A 5,000 DPM/μl ^3^H-METH stock concentration was prepared. The white sides of the RED device wells were loaded with 300 μl 1x PBS, and the red sides were loaded with 10 μl of ^3^H-METH, 40 μl of diluted scFv and 50 μl of quarter log dilutions, from 0.1 to 1000 nM, of unlabeled METH. RED plates were covered and rotated slowly on orbital shaker at room temperature overnight. Fifty microliters from each side of the RED device wells were sampled and added to scintillation vials containing 4 mL of ScintiVerse BD Cocktail. Vials were vortexed for 10 sec before the DPM were quantitated using a Tri-Carb 2910 TR scintillation counter. The percent of ^3^H-METH bound scFv was determined (DPM red side–DPM white side) / (DPM red side) x 100%. The percent ^3^H-METH bound was normalized, and the dilutions log_10_ transformed. The log_10_ of the IC_50_ was determined using one site fit nonlinear regression from the resulting data using GraphPad Prism (v6f, La Jolla, CA).

### Disposition of METH and AMP after AAV-scFv treatment

Thirty male BALB/c mice (10 mice per group) were injected with either 1x10^12^ vg/mouse AAV-scFv6H4, AAV-scFv7F9, or 1x PBS/5% sorbitol as described above. Blood samples were collected weekly, starting one week after injection, at a volume of 75 μL via tail snips to determine AAV-scFv concentration. Based on previous data (see Fig 3), peak serum concentrations occur between weeks two and three. The three treatment groups were subdivided into three time point groups, 30, 60, and 120 min. On Day 21 post-AAV-scFv injection, mice were given 0.56 mg/kg of METH via ip injection and then sacrificed at one of the three time points. Whole blood, serum, and organs were harvested and later analyzed via LC/MS-MS to determine METH concentration and partitioning in mouse brain and serum. Serum samples required no processing after separation from whole blood before LC/MS-MS analysis. Brains were homogenized in LC/MS-MS grade water at a ratio of 1:4. Homogenized samples were then centrifuged at 4000 x g for 20 min and the supernatant removed for analysis.

In a separate experiment, 30 male BALB/c mice were injected with either 1x10^12^ vg/mouse AAV-scFv or 1x PBS/5% sorbitol as described earlier. Blood samples were collected every other week to monitor expression levels. On day 50, a pharmacodynamics study was performed. Mice were injected *sc* with 1 mg/kg METH, and blood samples were collected at 30, 60, 120, and 180 min post METH treatment. Blood samples were centrifuged, and the resultant serum layer was stored at -80°C for later analysis via LC/MS-MS.

METH serum and brain concentrations were analyzed using LC/MS-MS as described previously.[26,52] Strata X-C 33 μM Polymeric Strong Cation solid phase extraction columns (Phenomenex, Torrance, CA) were used for the extraction method. METH/AMP standards, at concentrations of 0.3, 1, 3, 10, 30, 100, 300, 1000, and 2000 ng/mL, and quality control (QC) standards of 3, 10, and 800 ng/mL were used. The concentration of METH in serum samples and standards was determined using an Acquity Ultra Performance Liquid Chromatography system (Acquity UPLC BEH C18 1.7 μm column) and Quattro Premier XE mass spectrometer (Waters Corporation, Milford, MA). The lower and upper limit of quantification for METH was 1 and 2000 ng/mL, respectively, and 1 and 1000 ng/mL, respectively, for AMP. All standard values used in the calibration curves were within ± 20% of predicted concentrations. At least four of six of the QC standards total and half at each concentration were ± 20% of predicted concentrations.

### Statistical analyses

All data were analyzed using GraphPad Prism. Power analyses were performed to determine appropriate n values for each study using the http://clincalc.com/Stats/SampleSize.aspx site with power set to 0.8 and α = 0.05. Prior to analysis, any outliers were identified in the data using a Grubb’s outlier test. Data was tested for normality using Shapiro-Wilk normality tests. Variances were assumed to be similar. The resulting data from the hydrodynamic plasmid delivery study, from the METH biodistribution study, and from health monitoring were analyzed via two-way ANOVA using a Dunnett’s correction for multiple comparisons when a significant difference was found between groups. Linear regression of absorbance of standards with interpolation for unknowns was used to calculate expressed AAV-scFv concentrations. IC_50_s were calculated using either one-phase decay or non-linear one-site fit regression. For animal health studies, the data was analyzed via a two-way ANOVA. Since there was not enough serum to measure all three parameters for all animals at every sampling point, we could not use repeated-measures ANOVA. Significance was determined at p < 0.05 and denoted with *. Signicance of p < 0.01 denoted with #.

## Supporting information

S1 Fig Mouse body weights over time in control and AAV-scFv treated groups. There was no difference in rate of weight gain between groups over time based on a comparison of slope values (p > 0.2). Data points are mean ± SEM (n = 9–10 per group).
(EPS)

S2 Fig Changes in biochemical markers of liver and kidney health status over time. The above figures illustrate the changes in kidney (a. creatinine) and liver (b. ALT and c. AST) health over the second half of this long-term expression experiment. The horizontal dotted lines represent high and low normal values. Some sporadic significant differences between sham and treatment groups are observed (AST: sham v AAV-scFv7F9, days 138 and 149; creatinine: sham v AAV-scFv6H4, day 121) (* p < 0.05; #, p < 0.01). However, values remained at or below normal ranges. In the case where creatinine levels in the AAV-scFv6H4 group was higher than sham, it was not significantly higher than the normal high value (t-test, p = 0.8). Points are mean ± SEM (n = 6–8 per group).
(EPS)

S3 Fig Comparison of organ weights at 212 days post AAV-scFv injection. The liver weights were significantly different between sham and AAV-scFv6H4 groups only (*p < 0.05). All other organs were not significantly different between groups. Bars are mean ± SEM (n = 6–8 per group).
(EPS)

S1 Dataset (XLSX)
